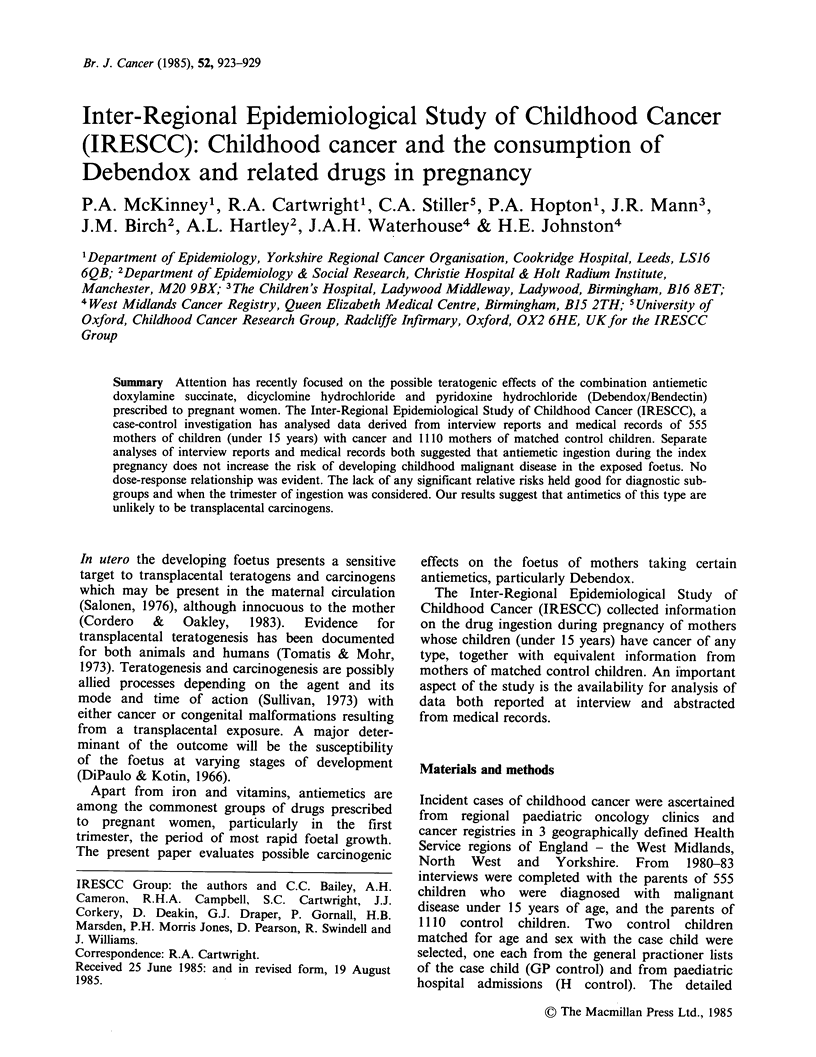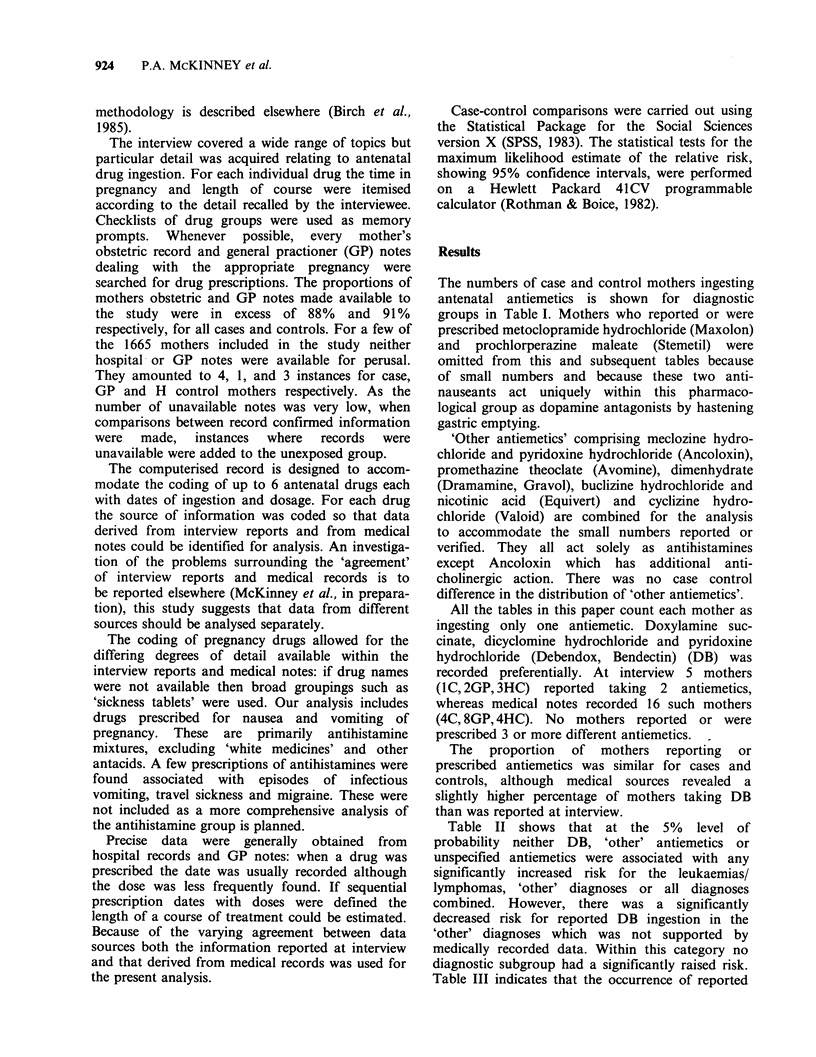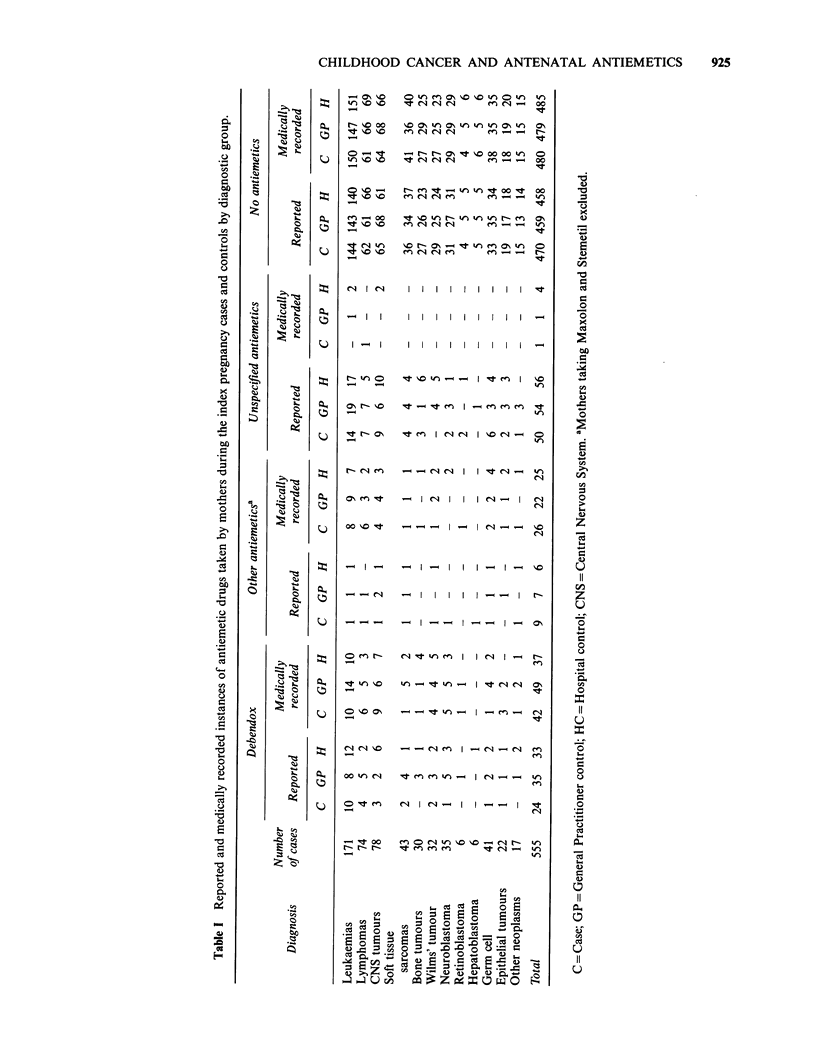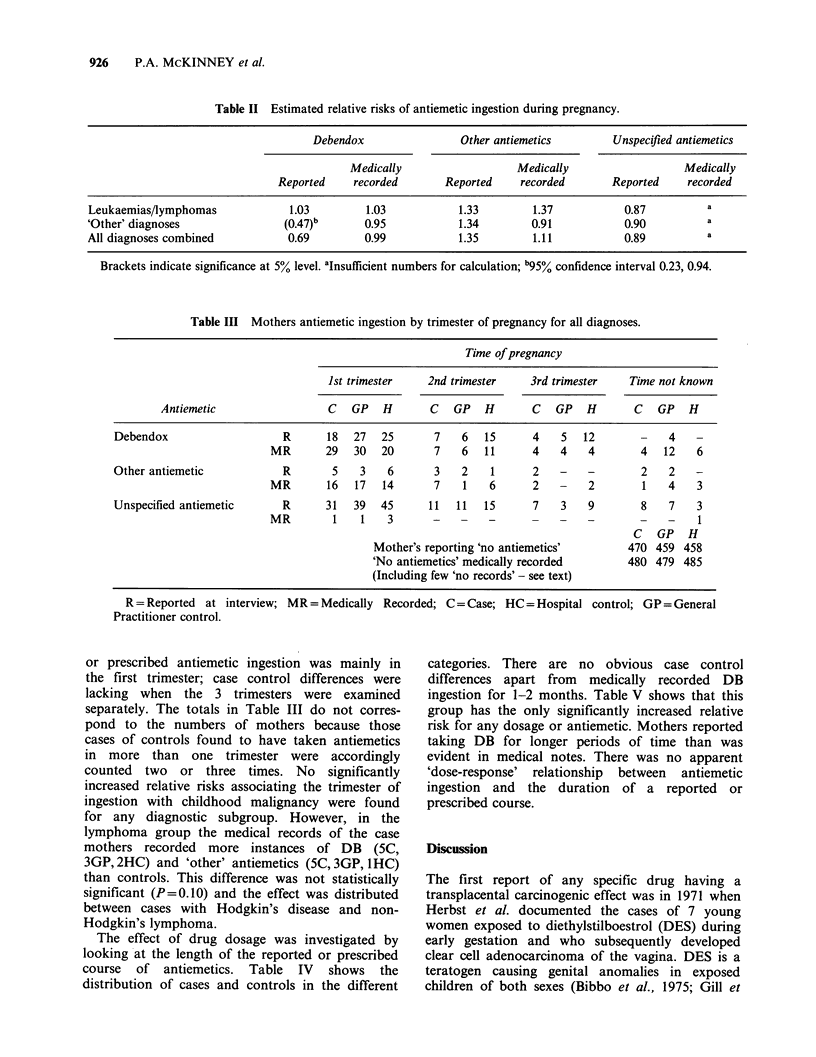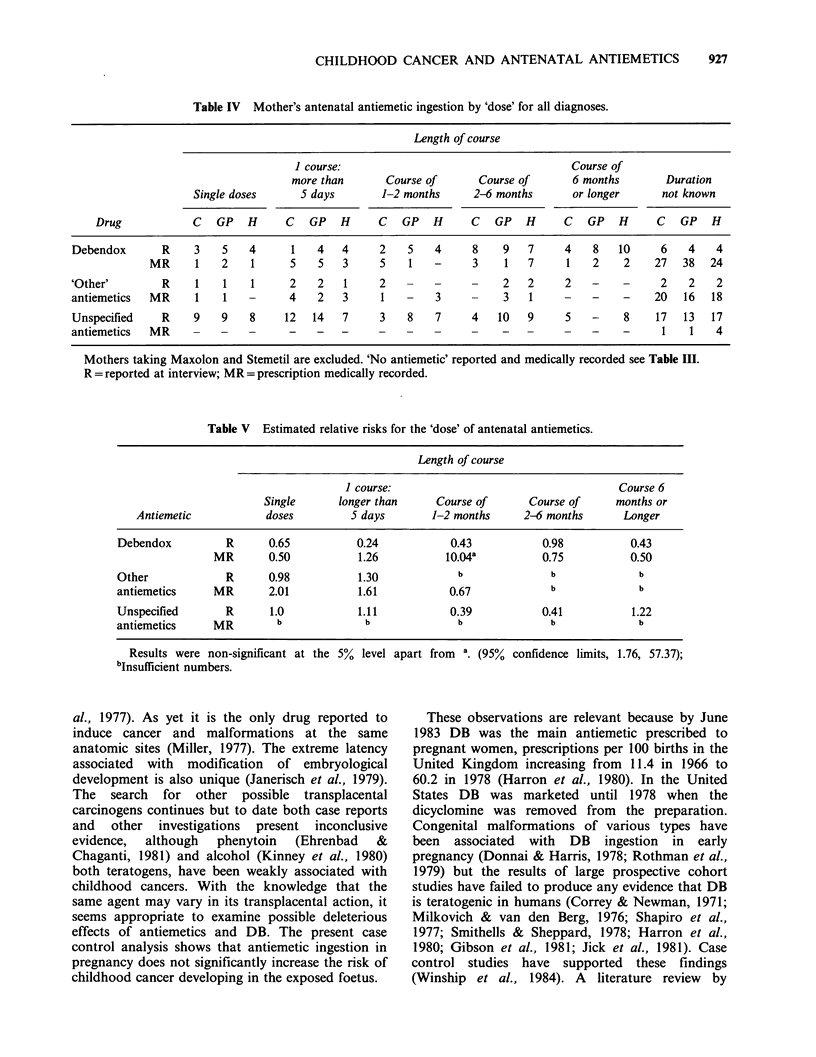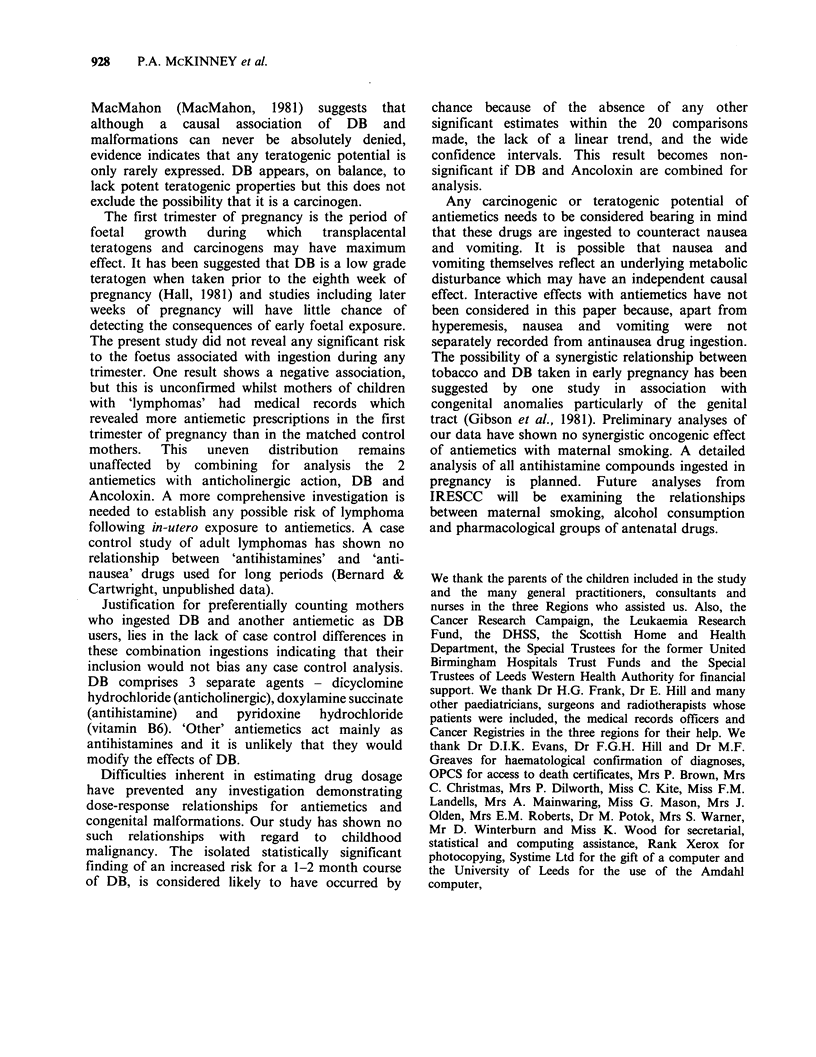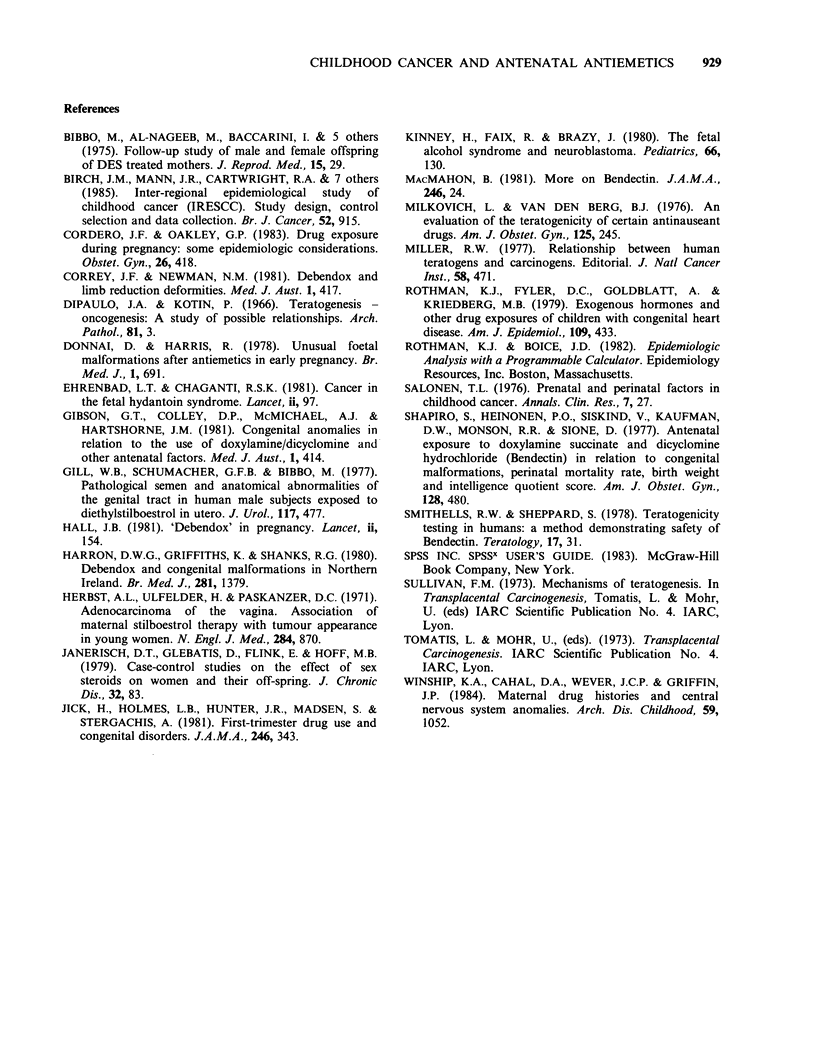# Inter-Regional Epidemiological Study of Childhood Cancer (IRESCC): childhood cancer and the consumption of debendox and related drugs in pregnancy.

**DOI:** 10.1038/bjc.1985.278

**Published:** 1985-12

**Authors:** P. A. McKinney, R. A. Cartwright, C. A. Stiller, P. A. Hopton, J. R. Mann, J. M. Birch, A. L. Hartley, J. A. Waterhouse, H. E. Johnston

## Abstract

Attention has recently focused on the possible teratogenic effects of the combination antiemetic doxylamine succinate, dicyclomine hydrochloride and pyridoxine hydrochloride (Debendox/Bendectin) prescribed to pregnant women. The Inter-Regional Epidemiological Study of Childhood Cancer (IRESCC), a case-control investigation has analysed data derived from interview reports and medical records of 555 mothers of children (under 15 years) with cancer and 1110 mothers of matched control children. Separate analyses of interview reports and medical records both suggested that antiemetic ingestion during the index pregnancy does not increase the risk of developing childhood malignant disease in the exposed foetus. No dose-response relationship was evident. The lack of any significant relative risks held good for diagnostic sub-groups and when the trimester of ingestion was considered. Our results suggest that antimetics of this type are unlikely to be transplacental carcinogens.


Br. J. Cancer (1985), 52, 923–929

### Inter-Regional Epidemiological Study of Childhood Cancer (IRESCC): Childhood cancer and the consumption of Debendox and related drugs in pregnancy

P.A. McKinney[1], R.A. Cartwright[1], C.A. Stiller[5], P.A. Hopton[1], J.R. Mann[3], J.M. Birch[2], A.L. Hartley[2], J.A.H. Waterhouse[4] & H.E. Johnston[4]

[1]*Department of Epidemiology, Yorkshire Regional Cancer Organisation, Cookridge Hospital, Leeds, LS16 6QB;* [2]*Department of Epidemiology & Social Research, Christie Hospital & Holt Radium Institute, Manchester, M20 9BX;* [3]*The Children's Hospital, Ladywood Middleway, Ladywood, Birmingham, B16 8ET;* [4]*West Midlands Cancer Registry, Queen Elizabeth Medical Centre, Birmingham, B15 2TH;* [5]*University of Oxford, Childhood Cancer Research Group, Radcliffe Infirmary, Oxford, OX2 6HE, UK for the IRESCC Group*

**Summary** Attention has recently focused on the possible teratogenic effects of the combination antiemetic doxylamine succinate, dicyclomine hydrochloride and pyridoxine hydrochloride (Debendox/Bendectin) prescribed to pregnant women. The Inter-Regional Epidemiological Study of Childhood Cancer (IRESCC), a case-control investigation has analysed data derived from interview reports and medical records of 555 mothers of children (under 15 years) with cancer and 1110 mothers of matched control children. Separate analyses of interview reports and medical records both suggested that antiemetic ingestion during the index pregnancy does not increase the risk of developing childhood malignant disease in the exposed foetus. No dose-response relationship was evident. The lack of any significant relative risks held good for diagnostic sub-groups and when the trimester of ingestion was considered. Our results suggest that antimetics of this type are unlikely to be transplacental carcinogens.

*In utero* the developing foetus presents a sensitive target to transplacental teratogens and carcinogens which may be present in the maternal circulation (Salonen, 1976), although innocuous to the mother (Cordero & Oakley, 1983). Evidence for transplacental teratogenesis has been documented for both animals and humans (Tomatis & Mohr, 1973). Teratogenesis and carcinogenesis are possibly allied processes depending on the agent and its mode and time of action (Sullivan, 1973) with either cancer or congenital malformations resulting from a transplacental exposure. A major determinant of the outcome will be the susceptibility of the foetus at varying stages of development (DiPaulo & Kotin, 1966).

Apart from iron and vitamins, antiemetics are among the commonest groups of drugs prescribed to pregnant women, particularly in the first trimester, the period of most rapid foetal growth. The present paper evaluates possible carcinogenic effects on the foetus of mothers taking certain antiemetics, particularly Debendox.

The Inter-Regional Epidemiological Study of Childhood Cancer (IRESCC) collected information on the drug ingestion during pregnancy of mothers whose children (under 15 years) have cancer of any type, together with equivalent information from mothers of matched control children. An important aspect of the study is the availability for analysis of data both reported at interview and abstracted from medical records.

### Materials and methods

Incident cases of childhood cancer were ascertained from regional paediatric oncology clinics and cancer registries in 3 geographically defined Health Service regions of England – the West Midlands, North West and Yorkshire. From 1980–83 interviews were completed with the parents of 555 children who were diagnosed with malignant disease under 15 years of age, and the parents of 1110 control children. Two control children matched for age and sex with the case child were selected, one each from the general practioner lists of the case child (GP control) and from paediatric hospital admissions (H control). The detailed

IRESCC Group: the authors and C.C. Bailey, A.H. Cameron, R.H.A. Campbell, S.C. Cartwright, J.J. Corkery, D. Deakin, G.J. Draper, P. Gornall, H.B. Marsden, P.H. Morris Jones, D. Pearson, R. Swindell and J. Williams.

Correspondence: R.A. Cartwright.

Received 25 June 1985: and in revised form, 19 August 1985.

© The Macmillan Press Ltd., 1985

methodology is described elsewhere (Birch *et al.*, 1985).

The interview covered a wide range of topics but particular detail was acquired relating to antenatal drug ingestion. For each individual drug the time in pregnancy and length of course were itemised according to the detail recalled by the interviewee. Checklists of drug groups were used as memory prompts. Whenever possible, every mother's obstetric record and general practioner (GP) notes dealing with the appropriate pregnancy were searched for drug prescriptions. The proportions of mothers obstetric and GP notes made available to the study were in excess of 88% and 91% respectively, for all cases and controls. For a few of the 1665 mothers included in the study neither hospital or GP notes were available for perusal. They amounted to 4, 1, and 3 instances for case, GP and H control mothers respectively. As the number of unavailable notes was very low, when comparisons between record confirmed information were made, instances where records were unavailable were added to the unexposed group.

The computerised record is designed to accommodate the coding of up to 6 antenatal drugs each with dates of ingestion and dosage. For each drug the source of information was coded so that data derived from interview reports and from medical notes could be identified for analysis. An investigation of the problems surrounding the 'agreement' of interview reports and medical records is to be reported elsewhere (McKinney *et al.*, in preparation), this study suggests that data from different sources should be analysed separately.

The coding of pregnancy drugs allowed for the differing degrees of detail available within the interview reports and medical notes: if drug names were not available then broad groupings such as 'sickness tablets' were used. Our analysis includes drugs prescribed for nausea and vomiting of pregnancy. These are primarily antihistamine mixtures, excluding 'white medicines' and other antacids. A few prescriptions of antihistamines were found associated with episodes of infectious vomiting, travel sickness and migraine. These were not included as a more comprehensive analysis of the antihistamine group is planned.

Precise data were generally obtained from hospital records and GP notes: when a drug was prescribed the date was usually recorded although the dose was less frequently found. If sequential prescription dates with doses were defined the length of a course of treatment could be estimated. Because of the varying agreement between data sources both the information reported at interview and that derived from medical records was used for the present analysis.

Case-control comparisons were carried out using the Statistical Package for the Social Sciences version X (SPSS, 1983). The statistical tests for the maximum likelihood estimate of the relative risk, showing 95% confidence intervals, were performed on a Hewlett Packard 41CV programmable calculator (Rothman & Boice, 1982).

### Results

The numbers of case and control mothers ingesting antenatal antiemetics is shown for diagnostic groups in Table I. Mothers who reported or were prescribed metoclopramide hydrochloride (Maxolon) and prochlorperazine maleate (Stemetil) were omitted from this and subsequent tables because of small numbers and because these two anti-nauseants act uniquely within this pharmacological group as dopamine antagonists by hastening gastric emptying.

'Other antiemetics' comprising meclozine hydrochloride and pyridoxine hydrochloride (Ancoloxin), promethazine theoclate (Avomine), dimenhydrate (Dramamine, Gravol), buclizine hydrochloride and nicotinic acid (Equivert) and cyclizine hydrochloride (Valoid) are combined for the analysis to accommodate the small numbers reported or verified. They all act solely as antihistamines except Ancoloxin which has additional anti-cholinergic action. There was no case control difference in the distribution of 'other antiemetics'.

All the tables in this paper count each mother as ingesting only one antiemetic. Doxylamine succinate, dicyclomine hydrochloride and pyridoxine hydrochloride (Debendox, Bendectin) (DB) was recorded preferentially. At interview 5 mothers (1C, 2GP, 3HC) reported taking 2 antiemetics, whereas medical notes recorded 16 such mothers (4C, 8GP, 4HC). No mothers reported or were prescribed 3 or more different antiemetics.

The proportion of mothers reporting or prescribed antiemetics was similar for cases and controls, although medical sources revealed a slightly higher percentage of mothers taking DB than was reported at interview.

Table II shows that at the 5% level of probability neither DB, 'other' antiemetics or unspecified antiemetics were associated with any significantly increased risk for the leukaemias/lymphomas, 'other' diagnoses or all diagnoses combined. However, there was a significantly decreased risk for reported DB ingestion in the 'other' diagnoses which was not supported by medically recorded data. Within this category no diagnostic subgroup had a significantly raised risk. Table III indicates that the occurrence of reported

**Table I** Reported and medically recorded instances of antiemetic drugs taken by mothers during the index pregnancy cases and controls by diagnostic group.

| Diagnosis | Number of cases | Debendox | | | | | | Other antiemetics[a] | | | | | | Unspecified antiemetics | | | | | | No antiemetics | | | | | |
|---|---|---|---|---|---|---|---|---|---|---|---|---|---|---|---|---|---|---|---|---|---|---|---|---|---|
| | | Reported | | | Medically recorded | | | Reported | | | Medically recorded | | | Reported | | | Medically recorded | | | Reported | | | Medically recorded | | |
| | | *C* | *GP* | *H* | *C* | *GP* | *H* | *C* | *GP* | *H* | *C* | *GP* | *H* | *C* | *GP* | *H* | *C* | *GP* | *H* | *C* | *GP* | *H* | *C* | *GP* | *H* |
| Leukaemias | 171 | 10 | 8 | 12 | 10 | 14 | 10 | 1 | 1 | 1 | 8 | 9 | 7 | 14 | 19 | 17 | – | 1 | 2 | 144 | 143 | 140 | 150 | 147 | 151 |
| Lymphomas | 74 | 4 | 5 | 2 | 6 | 5 | 3 | 1 | 1 | – | 6 | 3 | 2 | 7 | 7 | 5 | 1 | – | – | 62 | 61 | 66 | 61 | 66 | 69 |
| CNS tumours | 78 | 3 | 2 | 6 | 9 | 6 | 7 | 1 | 2 | 1 | 4 | 4 | 3 | 9 | 6 | 10 | – | – | 2 | 65 | 68 | 61 | 64 | 68 | 66 |
| Soft tissue sarcomas | 43 | 2 | 4 | 1 | 1 | 5 | 2 | 1 | 1 | 1 | 1 | 1 | 1 | 4 | 4 | 4 | – | – | – | 36 | 34 | 37 | 41 | 36 | 40 |
| Bone tumours | 30 | – | 3 | 1 | 1 | 1 | 4 | – | – | – | 1 | – | 1 | 3 | 1 | 6 | – | – | – | 27 | 26 | 23 | 27 | 29 | 25 |
| Wilms' tumour | 32 | 2 | 3 | 2 | 4 | 4 | 5 | 1 | – | 1 | 1 | 2 | 2 | – | 4 | 5 | – | – | – | 29 | 25 | 24 | 27 | 25 | 23 |
| Neuroblastoma | 35 | 1 | 5 | 3 | 5 | 5 | 3 | 1 | – | – | – | – | 2 | 2 | 3 | 1 | – | – | – | 31 | 27 | 31 | 29 | 29 | 29 |
| Retinoblastoma | 6 | – | 1 | – | 1 | 1 | – | – | – | – | 1 | – | – | 2 | – | 1 | – | – | – | 4 | 5 | 5 | 4 | 5 | 6 |
| Hepatoblastoma | 6 | – | – | 1 | – | – | – | 1 | – | – | – | – | – | – | 1 | – | – | – | – | 5 | 5 | 5 | 6 | 5 | 6 |
| Germ cell | 41 | 1 | 2 | 2 | 1 | 4 | 2 | 1 | 1 | 1 | 2 | 2 | 4 | 6 | 3 | 4 | – | – | – | 33 | 35 | 34 | 38 | 35 | 35 |
| Epithelial tumours | 22 | 1 | 1 | 1 | 3 | 2 | – | – | 1 | – | 1 | 1 | 2 | 2 | 3 | 3 | – | – | – | 19 | 17 | 18 | 18 | 19 | 20 |
| Other neoplasms | 17 | – | 1 | 2 | 1 | 2 | 1 | 1 | – | 1 | 1 | – | 1 | 1 | 3 | – | – | – | – | 15 | 13 | 14 | 15 | 15 | 15 |
| *Total* | 555 | 24 | 35 | 33 | 42 | 49 | 37 | 9 | 7 | 6 | 26 | 22 | 25 | 50 | 54 | 56 | 1 | 1 | 4 | 470 | 459 | 458 | 480 | 479 | 485 |

C = Case; GP = General Practitioner control; HC = Hospital control; CNS = Central Nervous System. [a]Mothers taking Maxolon and Stemetil excluded.

**Table II** Estimated relative risks of antiemetic ingestion during pregnancy.

| | *Debendox* | | *Other antiemetics* | | *Unspecified antiemetics* | |
|---|---|---|---|---|---|---|
| | *Reported* | *Medically recorded* | *Reported* | *Medically recorded* | *Reported* | *Medically recorded* |
| Leukaemias/lymphomas | 1.03 | 1.03 | 1.33 | 1.37 | 0.87 | [a] |
| 'Other' diagnoses | (0.47)[b] | 0.95 | 1.34 | 0.91 | 0.90 | [a] |
| All diagnoses combined | 0.69 | 0.99 | 1.35 | 1.11 | 0.89 | [a] |

Brackets indicate significance at 5% level. [a]Insufficient numbers for calculation; [b]95% confidence interval 0.23, 0.94.

**Table III** Mothers antiemetic ingestion by trimester of pregnancy for all diagnoses.

| | | *Time of pregnancy* | | | | | | | | | | | |
|---|---|---|---|---|---|---|---|---|---|---|---|---|---|
| | | *1st trimester* | | | *2nd trimester* | | | *3rd trimester* | | | *Time not known* | | |
| *Antiemetic* | | *C* | *GP* | *H* | *C* | *GP* | *H* | *C* | *GP* | *H* | *C* | *GP* | *H* |
| Debendox | R | 18 | 27 | 25 | 7 | 6 | 15 | 4 | 5 | 12 | – | 4 | – |
| | MR | 29 | 30 | 20 | 7 | 6 | 11 | 4 | 4 | 4 | 4 | 12 | 6 |
| Other antiemetic | R | 5 | 3 | 6 | 3 | 2 | 1 | 2 | – | – | 2 | 2 | – |
| | MR | 16 | 17 | 14 | 7 | 1 | 6 | 2 | – | 2 | 1 | 4 | 3 |
| Unspecified antiemetic | R | 31 | 39 | 45 | 11 | 11 | 15 | 7 | 3 | 9 | 8 | 7 | 3 |
| | MR | 1 | 1 | 3 | – | – | – | – | – | – | – | – | 1 |
| | | | | | | | | | | | *C* | *GP* | *H* |
| Mother's reporting 'no antiemetics' | | | | | | | | | | | 470 | 459 | 458 |
| 'No antiemetics' medically recorded (Including few 'no records' – see text) | | | | | | | | | | | 480 | 479 | 485 |

R = Reported at interview; MR = Medically Recorded; C = Case; HC = Hospital control; GP = General Practitioner control.

or prescribed antiemetic ingestion was mainly in the first trimester; case control differences were lacking when the 3 trimesters were examined separately. The totals in Table III do not correspond to the numbers of mothers because those cases of controls found to have taken antiemetics in more than one trimester were accordingly counted two or three times. No significantly increased relative risks associating the trimester of ingestion with childhood malignancy were found for any diagnostic subgroup. However, in the lymphoma group the medical records of the case mothers recorded more instances of DB (5C, 3GP, 2HC) and 'other' antiemetics (5C, 3GP, 1HC) than controls. This difference was not statistically significant ($P = 0.10$) and the effect was distributed between cases with Hodgkin's disease and non-Hodgkin's lymphoma.

The effect of drug dosage was investigated by looking at the length of the reported or prescribed course of antiemetics. Table IV shows the distribution of cases and controls in the different categories. There are no obvious case control differences apart from medically recorded DB ingestion for 1–2 months. Table V shows that this group has the only significantly increased relative risk for any dosage or antiemetic. Mothers reported taking DB for longer periods of time than was evident in medical notes. There was no apparent 'dose-response' relationship between antiemetic ingestion and the duration of a reported or prescribed course.

### Discussion

The first report of any specific drug having a transplacental carcinogenic effect was in 1971 when Herbst *et al.* documented the cases of 7 young women exposed to diethylstilboestrol (DES) during early gestation and who subsequently developed clear cell adenocarcinoma of the vagina. DES is a teratogen causing genital anomalies in exposed children of both sexes (Bibbo *et al.*, 1975; Gill *et*

**Table IV** Mother's antenatal antiemetic ingestion by 'dose' for all diagnoses.

| Drug | | Single doses | | | 1 course: more than 5 days | | | Course of 1–2 months | | | Course of 2–6 months | | | Course of 6 months or longer | | | Duration not known | | |
|---|---|---|---|---|---|---|---|---|---|---|---|---|---|---|---|---|---|---|---|
| | | C | GP | H | C | GP | H | C | GP | H | C | GP | H | C | GP | H | C | GP | H |
| Debendox | R | 3 | 5 | 4 | 1 | 4 | 4 | 2 | 5 | 4 | 8 | 9 | 7 | 4 | 8 | 10 | 6 | 4 | 4 |
| | MR | 1 | 2 | 1 | 5 | 5 | 3 | 5 | 1 | – | 3 | 1 | 7 | 1 | 2 | 2 | 27 | 38 | 24 |
| 'Other' | R | 1 | 1 | 1 | 2 | 2 | 1 | 2 | – | – | – | 2 | 2 | 2 | – | – | 2 | 2 | 2 |
| antiemetics | MR | 1 | 1 | – | 4 | 2 | 3 | 1 | – | 3 | – | 3 | 1 | – | – | – | 20 | 16 | 18 |
| Unspecified | R | 9 | 9 | 8 | 12 | 14 | 7 | 3 | 8 | 7 | 4 | 10 | 9 | 5 | – | 8 | 17 | 13 | 17 |
| antiemetics | MR | – | – | – | – | – | – | – | – | – | – | – | – | – | – | – | 1 | 1 | 4 |

Mothers taking Maxolon and Stemetil are excluded. 'No antiemetic' reported and medically recorded see **Table III**.
R = reported at interview; MR = prescription medically recorded.

**Table V** Estimated relative risks for the 'dose' of antenatal antiemetics.

| Antiemetic | | Single doses | 1 course: longer than 5 days | Course of 1–2 months | Course of 2–6 months | Course 6 months or Longer |
|---|---|---|---|---|---|---|
| Debendox | R | 0.65 | 0.24 | 0.43 | 0.98 | 0.43 |
| | MR | 0.50 | 1.26 | 10.04[a] | 0.75 | 0.50 |
| Other | R | 0.98 | 1.30 | [b] | [b] | [b] |
| antiemetics | MR | 2.01 | 1.61 | 0.67 | [b] | [b] |
| Unspecified | R | 1.0 | 1.11 | 0.39 | 0.41 | 1.22 |
| antiemetics | MR | [b] | [b] | [b] | [b] | [b] |

Results were non-significant at the 5% level apart from [a]. (95% confidence limits, 1.76, 57.37); [b]Insufficient numbers.

*al.*, 1977). As yet it is the only drug reported to induce cancer and malformations at the same anatomic sites (Miller, 1977). The extreme latency associated with modification of embryological development is also unique (Janerisch *et al.*, 1979). The search for other possible transplacental carcinogens continues but to date both case reports and other investigations present inconclusive evidence, although phenytoin (Ehrenbad & Chaganti, 1981) and alcohol (Kinney *et al.*, 1980) both teratogens, have been weakly associated with childhood cancers. With the knowledge that the same agent may vary in its transplacental action, it seems appropriate to examine possible deleterious effects of antiemetics and DB. The present case control analysis shows that antiemetic ingestion in pregnancy does not significantly increase the risk of childhood cancer developing in the exposed foetus.

These observations are relevant because by June 1983 DB was the main antiemetic prescribed to pregnant women, prescriptions per 100 births in the United Kingdom increasing from 11.4 in 1966 to 60.2 in 1978 (Harron *et al.*, 1980). In the United States DB was marketed until 1978 when the dicyclomine was removed from the preparation. Congenital malformations of various types have been associated with DB ingestion in early pregnancy (Donnai & Harris, 1978; Rothman *et al.*, 1979) but the results of large prospective cohort studies have failed to produce any evidence that DB is teratogenic in humans (Correy & Newman, 1971; Milkovich & van den Berg, 1976; Shapiro *et al.*, 1977; Smithells & Sheppard, 1978; Harron *et al.*, 1980; Gibson *et al.*, 1981; Jick *et al.*, 1981). Case control studies have supported these findings (Winship *et al.*, 1984). A literature review by

MacMahon (MacMahon, 1981) suggests that although a causal association of DB and malformations can never be absolutely denied, evidence indicates that any teratogenic potential is only rarely expressed. DB appears, on balance, to lack potent teratogenic properties but this does not exclude the possibility that it is a carcinogen.

The first trimester of pregnancy is the period of foetal growth during which transplacental teratogens and carcinogens may have maximum effect. It has been suggested that DB is a low grade teratogen when taken prior to the eighth week of pregnancy (Hall, 1981) and studies including later weeks of pregnancy will have little chance of detecting the consequences of early foetal exposure. The present study did not reveal any significant risk to the foetus associated with ingestion during any trimester. One result shows a negative association, but this is unconfirmed whilst mothers of children with 'lymphomas' had medical records which revealed more antiemetic prescriptions in the first trimester of pregnancy than in the matched control mothers. This uneven distribution remains unaffected by combining for analysis the 2 antiemetics with anticholinergic action, DB and Ancoloxin. A more comprehensive investigation is needed to establish any possible risk of lymphoma following *in-utero* exposure to antiemetics. A case control study of adult lymphomas has shown no relationship between 'antihistamines' and 'anti-nausea' drugs used for long periods (Bernard & Cartwright, unpublished data).

Justification for preferentially counting mothers who ingested DB and another antiemetic as DB users, lies in the lack of case control differences in these combination ingestions indicating that their inclusion would not bias any case control analysis. DB comprises 3 separate agents – dicyclomine hydrochloride (anticholinergic), doxylamine succinate (antihistamine) and pyridoxine hydrochloride (vitamin B6). 'Other' antiemetics act mainly as antihistamines and it is unlikely that they would modify the effects of DB.

Difficulties inherent in estimating drug dosage have prevented any investigation demonstrating dose-response relationships for antiemetics and congenital malformations. Our study has shown no such relationships with regard to childhood malignancy. The isolated statistically significant finding of an increased risk for a 1–2 month course of DB, is considered likely to have occurred by chance because of the absence of any other significant estimates within the 20 comparisons made, the lack of a linear trend, and the wide confidence intervals. This result becomes non-significant if DB and Ancoloxin are combined for analysis.

Any carcinogenic or teratogenic potential of antiemetics needs to be considered bearing in mind that these drugs are ingested to counteract nausea and vomiting. It is possible that nausea and vomiting themselves reflect an underlying metabolic disturbance which may have an independent causal effect. Interactive effects with antiemetics have not been considered in this paper because, apart from hyperemesis, nausea and vomiting were not separately recorded from antinausea drug ingestion. The possibility of a synergistic relationship between tobacco and DB taken in early pregnancy has been suggested by one study in association with congenital anomalies particularly of the genital tract (Gibson *et al.*, 1981). Preliminary analyses of our data have shown no synergistic oncogenic effect of antiemetics with maternal smoking. A detailed analysis of all antihistamine compounds ingested in pregnancy is planned. Future analyses from IRESCC will be examining the relationships between maternal smoking, alcohol consumption and pharmacological groups of antenatal drugs.

We thank the parents of the children included in the study and the many general practitioners, consultants and nurses in the three Regions who assisted us. Also, the Cancer Research Campaign, the Leukaemia Research Fund, the DHSS, the Scottish Home and Health Department, the Special Trustees for the former United Birmingham Hospitals Trust Funds and the Special Trustees of Leeds Western Health Authority for financial support. We thank Dr H.G. Frank, Dr E. Hill and many other paediatricians, surgeons and radiotherapists whose patients were included, the medical records officers and Cancer Registries in the three regions for their help. We thank Dr D.I.K. Evans, Dr F.G.H. Hill and Dr M.F. Greaves for haematological confirmation of diagnoses, OPCS for access to death certificates, Mrs P. Brown, Mrs C. Christmas, Mrs P. Dilworth, Miss C. Kite, Miss F.M. Landells, Mrs A. Mainwaring, Miss G. Mason, Mrs J. Olden, Mrs E.M. Roberts, Dr M. Potok, Mrs S. Warner, Mr D. Winterburn and Miss K. Wood for secretarial, statistical and computing assistance, Rank Xerox for photocopying, Systime Ltd for the gift of a computer and the University of Leeds for the use of the Amdahl computer,

### References

BIBBO, M., AL-NAGEEB, M., BACCARINI, I. & 5 others (1975). Follow-up study of male and female offspring of DES treated mothers. *J. Reprod. Med.*, **15**, 29.

BIRCH, J.M., MANN, J.R., CARTWRIGHT, R.A. & 7 others (1985). Inter-regional epidemiological study of childhood cancer (IRESCC). Study design, control selection and data collection. *Br. J. Cancer*, **52**, 915.

CORDERO, J.F. & OAKLEY, G.P. (1983). Drug exposure during pregnancy: some epidemiologic considerations. *Obstet. Gyn.*, **26**, 418.

CORREY, J.F. & NEWMAN, N.M. (1981). Debendox and limb reduction deformities. *Med. J. Aust.* **1**, 417.

DIPAULO, J.A. & KOTIN, P. (1966). Teratogenesis – oncogenesis: A study of possible relationships. *Arch. Pathol.*, **81**, 3.

DONNAI, D. & HARRIS, R. (1978). Unusual foetal malformations after antiemetics in early pregnancy. *Br. Med. J.*, **1**, 691.

EHRENBAD, L.T. & CHAGANTI, R.S.K. (1981). Cancer in the fetal hydantoin syndrome. *Lancet*, **ii**, 97.

GIBSON, G.T., COLLEY, D.P., McMICHAEL, A.J. & HARTSHORNE, J.M. (1981). Congenital anomalies in relation to the use of doxylamine/dicyclomine and other antenatal factors. *Med. J. Aust.*, **1**, 414.

GILL, W.B., SCHUMACHER, G.F.B. & BIBBO, M. (1977). Pathological semen and anatomical abnormalities of the genital tract in human male subjects exposed to diethylstilboestrol in utero. *J. Urol.*, **117**, 477.

HALL, J.B. (1981). 'Debendox' in pregnancy. *Lancet*, **ii**, 154.

HARRON, D.W.G., GRIFFITHS, K. & SHANKS, R.G. (1980). Debendox and congenital malformations in Northern Ireland. *Br. Med. J.*, **281**, 1379.

HERBST, A.L., ULFELDER, H. & PASKANZER, D.C. (1971). Adenocarcinoma of the vagina. Association of maternal stilboestrol therapy with tumour appearance in young women. *N. Engl. J. Med.*, **284**, 870.

JANERISCH, D.T., GLEBATIS, D., FLINK, E. & HOFF, M.B. (1979). Case-control studies on the effect of sex steroids on women and their off-spring. *J. Chronic Dis.*, **32**, 83.

JICK, H., HOLMES, L.B., HUNTER, J.R., MADSEN, S. & STERGACHIS, A. (1981). First-trimester drug use and congenital disorders. *J.A.M.A.*, **246**, 343.

KINNEY, H., FAIX, R. & BRAZY, J. (1980). The fetal alcohol syndrome and neuroblastoma. *Pediatrics*, **66**, 130.

MacMAHON, B. (1981). More on Bendectin. *J.A.M.A.*, **246**, 24.

MILKOVICH, L. & VAN DEN BERG, B.J. (1976). An evaluation of the teratogenicity of certain antinauseant drugs. *Am. J. Obstet. Gyn.*, **125**, 245.

MILLER, R.W. (1977). Relationship between human teratogens and carcinogens. Editorial. *J. Natl Cancer Inst.*, **58**, 471.

ROTHMAN, K.J., FYLER, D.C., GOLDBLATT, A. & KRIEDBERG, M.B. (1979). Exogenous hormones and other drug exposures of children with congenital heart disease. *Am. J. Epidemiol.*, **109**, 433.

ROTHMAN, K.J. & BOICE, J.D. (1982). *Epidemiologic Analysis with a Programmable Calculator*. Epidemiology Resources, Inc. Boston, Massachusetts.

SALONEN, T.L. (1976). Prenatal and perinatal factors in childhood cancer. *Annals. Clin. Res.*, **7**, 27.

SHAPIRO, S., HEINONEN, P.O., SISKIND, V., KAUFMAN, D.W., MONSON, R.R. & SIONE, D. (1977). Antenatal exposure to doxylamine succinate and dicyclomine hydrochloride (Bendectin) in relation to congenital malformations, perinatal mortality rate, birth weight and intelligence quotient score. *Am. J. Obstet. Gyn.*, **128**, 480.

SMITHELLS, R.W. & SHEPPARD, S. (1978). Teratogenicity testing in humans: a method demonstrating safety of Bendectin. *Teratology*, **17**, 31.

SPSS INC. SPSS$^x$ USER'S GUIDE. (1983). McGraw-Hill Book Company, New York.

SULLIVAN, F.M. (1973). Mechanisms of teratogenesis. In *Transplacental Carcinogenesis*, Tomatis, L. & Mohr, U. (eds) IARC Scientific Publication No. 4. IARC, Lyon.

TOMATIS, L. & MOHR, U., (eds). (1973). *Transplacental Carcinogenesis*. IARC Scientific Publication No. 4. IARC, Lyon.

WINSHIP, K.A., CAHAL, D.A., WEVER, J.C.P. & GRIFFIN, J.P. (1984). Maternal drug histories and central nervous system anomalies. *Arch. Dis. Childhood*, **59**, 1052.